# Chitosan-Coated Silver–Vancomycin Nanoparticles for Treatment of Bacterial Endophthalmitis

**DOI:** 10.1167/iovs.67.3.20

**Published:** 2026-03-10

**Authors:** Henry Kolge, Zeeshan Ahmad, Sukhvinder Singh, Michael Yu, Ashok Kumar

**Affiliations:** 1 Department of Ophthalmology, Visual and Anatomical Sciences, Wayne State University School of Medicine, Detroit, Michigan, United States; 2 Department of Biochemistry, Microbiology, and Immunology, Wayne State University School of Medicine, Detroit, Michigan, United States

**Keywords:** endophthalmitis, nanoparticles, bacteria, eye, retina

## Abstract

**Purpose:**

The rising incidence of drug-resistant ocular pathogens highlights the critical need for innovative therapeutic strategies. Here, we developed an antibacterial nanoformulation by combining silver with an antibiotic (vancomycin) and evaluated its efficacy using in vitro, ex vivo, and in vivo models of ocular *Staphylococcus* *aureus* infections.

**Methods:**

Silver nanoparticles were synthesized through chemical reduction using silver nitrate and sodium citrate, subsequently loaded with vancomycin, and coated with chitosan to impart a positive surface charge. These nanoformulations were tested for their antimicrobial activity against *S. aureus*, including methicillin-resistant *S. aureus* (MRSA) and clinical isolates. The toxicity was evaluated in cultured human retinal Müller glia cells and mouse eyes. Ex vivo studies were performed using porcine/goat eyes and human vitreous. Therapeutic efficacy was evaluated in a mouse model of *S. aureus* endophthalmitis.

**Results:**

Three formulations (AgNPs, CAgNPs, and CAgVNPs) ranged in size from 60 to 70 nm and demonstrated efficient vancomycin loading with controlled release in acidic conditions. Antimicrobial testing revealed synergistic antibacterial effects of nanoformulations against *S. aureus*, including MRSA and clinical isolates, resulting in a two- to fourfold reduction in minimum inhibitory concentration compared to vancomycin. Both in vitro and in vivo assays showed negligible cytotoxicity. All nanoformulations ameliorated the severity of *S. aureus* endophthalmitis, with CAgVNPs showing a marked reduction in bacterial burden (two- to sevenfold) and inflammatory cytokines (three- to fivefold). Moreover, CAgVNP-treated eyes exhibited reduced cell death and retinal tissue damage.

**Conclusions:**

Our study demonstrates that the synergistic activity of chitosan-coated silver nanoparticles and antibiotics represents a promising therapeutic strategy for treating resistant ocular bacterial infections.

Bacterial pathogens remain the leading cause of ocular infections, such as keratitis and endophthalmitis, which often lead to blindness if not diagnosed or treated promptly.^1^^,^^2^ Endophthalmitis of either form, exogenous or endogenous, can lead to a significant reduction of visual acuity and, at worst, result in a loss of the affected eye.^3^ Moreover, there is an increasing concern about treatment failure due to the emergence of antibiotic resistance among ocular bacterial pathogens.^4^ The ARMOR surveillance study indicates that half of all staphylococcal isolates from the vitreous and aqueous are methicillin-resistant,^5^ indicating potential challenges in their treatments.

The current treatment of bacterial endophthalmitis primarily involves intravitreal injection of broad-spectrum antibiotics, which allows for direct delivery of high drug concentrations to the vitreous cavity.^6^^,^^7^ In severe cases, adjunct surgical interventions such as vitrectomy are needed.^8^^,^^9^ However, these approaches present several challenges. The injection itself is an invasive procedure and, paradoxically, can sometimes be a source of endophthalmitis if aseptic technique is compromised.^10^^,^^11^ Additionally, some cases require repeated injections due to persistent infection or poor initial response, which increases the risk of complications such as retinal detachment or vitreous hemorrhage.^12^^,^^13^ Another major limitation is the difficulty in maintaining effective long-term antibiotic levels in the vitreous, as most antibiotics have limited half-lives in the intraocular environment.^14^ This necessitates frequent administration or alternative strategies, such as sustained-release drug delivery systems, which are still under development.^15^^,^^16^

In recent years, nanoparticles have garnered significant attention and research for their application as drug delivery vehicles. Their submicron size, editable zeta potential, unique physicochemical properties, and multiloading characteristics make nanoparticles a desirable vehicle to transport drugs and active compounds to cell-specific targets.^17^^,^^18^ Nanoparticles can be categorized into various types: polymeric, metallic, lipid, or hybrid mixed.^19^^,^^20^ Their capability to adsorb, entrap, or covalently bind to drugs or different molecules formulated via diverse techniques makes them a reliable delivery vehicle.^21^^,^^22^ Moreover, several biodegradable and biocompatible polymers are used to synthesize nanoparticles with dissolved, dispersed, or surface-bound drugs.^23^ Furthermore, nanoparticles can overcome physiological barriers that exist in various organs, including the eye.^24^ Studies have shown improvement in the nanoparticles’ precorneal contact time and transcorneal permeability properties to augment intraocular bioavailability.^25^ Once internalized by corneal epithelial cells, nanoparticles can act as a drug reservoir, enabling prolonged and controlled drug release.^26^ Similarly, both polymeric and lipid nanoparticles show great promise for delivering therapeutics to the anterior and posterior eye diseases.^23^ Simultaneously, sustained release of drugs, via these nano-vectors, averts rapid loss of the drug via nasolacrimal discharge and rapid tear turnover.^27^

Silver nanoparticles have been reported to show efficient antibacterial and antifungal activity against gram-positive and gram-negative bacteria and fungi. Studies suggest that silver (Ag) in nanoform is a more effective and biocompatible agent with higher antibacterial activity than free Ag^+^.^28^ Zwitterionic silver nanoparticles have been used against bacterial keratitis.^29^^,^^30^ Similarly, the chitosan polymer has been extensively used in drug delivery because of its desirable properties such as biocompatibility, nontoxicity, biodegradability, mucoadhesion, and ability to enhance cell permeability.^31^ Moreover, when used in nanoformulation, chitosan provides a higher surface-to-volume ratio, and because of its positive charge, it has increased affinity to bacterial and fungal cell walls, contributing to inherent antimicrobial activity.^32^

Our research interests focus on developing newer therapies, including nanotechnology-based therapies, used alone or in combination with antibiotics, for effective treatment of ocular infections.^33^ Leveraging the broad-spectrum antimicrobial activity of silver and the strong affinity of chitosan for microbial cell walls, we developed chitosan-based antimicrobial nanoformulations for ocular delivery. Because the synergistic use of silver and antibiotics in ocular infections has not been previously evaluated, we synthesized silver nanoparticles, combined them with an antibiotic, and encapsulated them within chitosan to enable sustained delivery. We then evaluated their toxicity and antibacterial efficacy against ocular bacterial pathogens using in vitro, ex vivo, and in vivo models.

## Materials and Methods

### Synthesis of Nanoparticles

Silver nanoparticles were synthesized via the chemical reduction method (Supplementary Fig. S1), wherein silver nitrate (0.5 µM; Thermo Fisher, Waltham, MA, USA) was reduced in the presence of sodium citrate (0.5 µM; Thermo Fisher).^34^ They were preliminarily characterized for the presence of the specific peak at a 430-nm absorbance. These silver nanoparticles were subsequently coated with chitosan polymer to enhance their adherence and make them positively charged. Chitosan solution (0.5 mg/mL dissolved in 0.1 M acetic acid; Sigma, St. Louis, MO, USA) was added dropwise to the nanoparticles under stirring at 1000 revolutions per minute (rpm) for 1 hour. These nanoparticles were extensively characterized for size and charge using a Malvern Zetasizer (Nano ZSP-90; Worcestershire, Nano, UK) and further for their shape by scanning electron microscopy (SEM; Zeiss EVO MA15; Oberkochen, Zeiss, Germany) and transmission electron microscopy (TEM; Thermo Fisher).^35^ Elemental characterization was carried out using Scanning Transmission electron microscopy (STEM; Thermo Fisher).

### Drug Loading and Entrapment

The optimized nanoparticles underwent drug loading with vancomycin, a known antibiotic for the treatment of endophthalmitis. For drug entrapment, vancomycin (5 mg/mL) was mixed with silver nanoparticles solution, and chitosan was added dropwise (Supplementary Fig. S1). Drug loading was estimated in the supernatant by measuring absorbance at 290 nm in a Biotek Synergy HT plate reader (Biotek, Winooski, VT, USA). The drug loading percent and weight percent loading were calculated according to Chopra et al.^36^

$$\begin{array}{ccc} & & \;\mathrm{Drug}\;\mathrm{Loading}\;\left(\%\right)\\ & & \;=\frac{\mathrm{Total}\;\mathrm{drug}\;\mathrm{used}-\mathrm{Drug}\;\mathrm{in}\;\mathrm{supernatant}}{\mathrm{Total}\;\mathrm{drug}\;\mathrm{used}}\times 100 \end{array}$$

$$\begin{array}{ccc} & & \;\mathrm{Drug}\;\mathrm{Loading}\;\left(\mathrm{Weight}\;\mathrm{percent}\right)\\ & & \;=\frac{\mathrm{Drug}\;\mathrm{loading}\;\left(\mathrm{weight}\right)}{\mathrm{Dry}\;\mathrm{weight}\;\mathrm{of}\;\mathrm{nanoformulation}}\times 100 \end{array}$$

Absorbance of 10 to 50 µg/mL vancomycin was measured at 290 nm to generate the standard graph. Vancomycin-loaded nanoparticles were dried for the calculation of the weight percent of drug in the nanoformulation. Drug-loaded nanoformulations were characterized for size, charge, and shape using the Zetasizer, SEM, and TEM. Drug loading on the nanoparticles was characterized by the presence of respective elements as observed through STEM analysis.

### In Vitro Drug Release

CAgVNPs were analyzed for their drug (vancomycin) release profiles at pH 4.0 and 7.0. The release assay was designed to assess the pH-responsive behavior of the nanoformulation at pH 7.0 (mimicking the normal intraocular milieu) and pH 4.0 (an acidic microenvironment due to infection). The nanoformulations (1 mg) were dispersed in 1 mL of acetate buffer (pH 4) and PBS (pH 7.0) and incubated at 37°C for 120 hours. Absorbance was recorded at 290 nm intermittently for 5 days in the supernatant, after centrifugation of nanoformulations at 12,000*g* for 5 minutes. Higuchi's model of drug release was also applied by plotting the cumulative drug release against the square root of time, and the Higuchi constant was collected. Q = K_H_ × t^1/2^, where K_H_ is the Higuchi dissolution constant, and Q is the cumulative percentage drug release at time t.^37^

### In Vitro Antibacterial Activity of Nanoformulations

Antibacterial assays were performed using the laboratory *Staphylococcus* *aureus* (RN6390), clinical endophthalmitis isolates, and methicillin-resistant strains of *S. aureus* (SA) according to Clinical and Laboratory Standards Institute guidelines (CLSI M02, M07, and M11) with minor modifications. Briefly, bacterial cultures were inoculated in tryptone soy broth 24 hours before the experiment. The next day, the bacterial culture was adjusted to 0.5 OD, and 10^4^ CFU of bacteria were inoculated into each well of a 96-well plate. The nanoformulations silver nanoparticles (AgNPs), chitosan coated silver nanoparticles (CAgNPs) and chitosan coated silver-vancomycin loaded nanoparticles (CAgVNPs) were used at 0.25 to 32 µg/mL with concentrations adjusted according to the drug (vancomycin) content. Plates were incubated for 24 hours, the absorbance was measured at 600 nm, and the minimum inhibitory concentration for inhibition the growth of 90% of bacteria (MIC-90) was calculated based on bacterial viability. Untreated bacteria and plain media were used as controls. Electron microscopy, including STEM, was performed to visualize the antibacterial activity of nanoformulations against *S. aureus*.

### Toxicity Studies of Nanoformulations Against Müller Glia Cells

The calcein-AM assay was performed to evaluate the toxicity of nanoformulations using the human retinal Müller glia cells (MIO M1 cell line). The cells were preinfected with *S. aureus* for 2 hours, followed by the nanoformulation treatment. After 6 hours, cells were treated with calcein-AM dye and analyzed by a live-cell imaging system, IncuCyte (Sartorius, Göttingen, Germany), in which the live cells emit green fluorescence. In addition, a lactate dehydrogenase assay (Fisher Scientific, Waltham MA, USA) was also performed to estimate the toxicity of the nanoformulations at 6 hours. An AlamarBlue (AB) assay was also performed to check cytotoxicity by measuring the metabolism of cells, that is, the conversion of resazurin (oxidized form, blue) to resorufin (reduced form, red). Briefly, cells were seeded in a 96-well plate and incubated with varying concentrations of drug and nanoformulations (2.5 to 80 µg/mL) for 24 and 48 hours. The AB reagent (Thermo Fisher) was added, the plate was incubated for 2 hours, and the color change was measured at 570 nm. Cell viability was calculated with respect to the untreated control cells.

### Cellular Uptake and Intracellular Antimicrobial Activity

Cellular uptake of nanoparticles was studied microscopically and estimated spectrophotometrically. Red fluorescent protein (RFP) expressing SA was used to infect the MIO M1 cells. After 2 hours of infection, the cells were treated with gentamicin (200 µg/mL) to remove the extracellular bacteria. After gentamicin treatment for an hour, the cells were washed in media and treated with the drug and nanoformulations (Van, CAgNPs, CAgVNPs; 0.5 µg/mL) for 4 hours. Cells were washed and fixed, and fluorescent microscopy was performed. The cellular uptake of nanoparticles was quantified by exposing MIO M1 cells to CAgNPs and checking their concentrations in culture supernatant and cell lysates at different time points (0, 30, 60, 120, and 240 minutes).

SEM was performed to analyze the effect of nanoformulations on SA-infected Müller glia and immortalized bone marrow–derived macrophages (iBMDMs). The cells were cultured on sterile coverslips in a 6-well plate and infected with SA for 2 hours. Afterward, cells were treated with vancomycin or CAgVNPs for 4 hours, rinsed with PBS, fixed with 4% paraformaldehyde (PFA), and examined using electron microscopy.

### Ex Vivo and Vitreous Antimicrobial Activity of Nanoformulations

Ex vivo experiments were performed on pig eyes (collected from a local butcher shop). The eyes were cleaned and sterilized in 5% povidone iodide solution. These eyes were intravitreally injected with 100 µL SA (5 × 10^6^ CFU, 0.5 OD) and incubated for 1 hour at 37°C. Afterward, CAgVNPs, CAgNPs, and vancomycin (1 µg/mL) were intravitreally injected in SA-infected pig eyes and incubated for 5 hours, the vitreous was surgically removed, and serial dilutions were performed and plated on the tryptic soy agar (TSA) plates.

The vitreous retrieved from the pig, goat, and human eyes was used to assess its effect on the antimicrobial properties of nanoformulations. Briefly, SA (5-µL volume of 0.5 OD, 2.5 × 10^5^ CFU) was inoculated in 100 µL of the pig or human vitreous in a 96-well plate and incubated on a rotational shaker (150 rpm) for 1 hour at 37°C. Afterward, CAgVNPs, CAgNPs, and vancomycin (1 µg/mL) were added, and the plates were incubated for another 5 hours. The live bacterial count was enumerated by serial dilution on TSA plates.

### Mice and Ethical Statement

C57BL/6 (B6) mice (male and female, aged 6–8 weeks) were purchased from the Jackson Laboratory (Bar Harbor, ME, USA). Mice were housed in a restricted-access Division of Laboratory Animal Resources facility at the Kresge Eye Institute, maintained on a 12-hour light/dark cycle at 22°C, and fed with a LabDiet rodent chow (PicoLab; LabDiet, St. Louis, MO, USA) and provided water ad libitum. All procedures were performed in compliance with the ARVO statement for the use of animals and were approved by the institutional animal care and use committee of Wayne State University.

### In Vivo Efficacy of Nanoformulations

In vivo efficacy of the nanoformulations was examined in a well-established mouse model of *S. aureus* endophthalmitis, wherein eyes of C57BL/6 were infected with 5000 CFU/eye of SA strain RN6390 by intravitreal injection in a 1.0-µL volume of PBS. Eyes injected with PBS served as controls.^38^^–^^40^ Six hours postinfection, various nanoformulations (CAgVNPs, CAgNPs, and vancomycin) were injected at a concentration of 0.2 µg/µL. Disease severity was assessed in a blinded manner using a slit-lamp examination and clinical scores ranging from 0 to 4 (ranging from 0 to 4, with 0 being the best and 4 being the worst) following various treatments per the previously described criteria.^41^ TUNEL assay and hematoxylin and eosin staining were performed for the histological evaluation and to determine retinal tissue damage. The whole eye lysates were used to determine bacterial burden by plate count. The tissue lysates were stored at −20°C and thawed immediately for protein estimation and cytokine ELISA.^42^

### Statistical Analysis

Data normality was checked via the Shapiro–Wilk test, and the data passed the normality test without the requirement of log-transformation. The data are represented as the mean ± SD. All statistical significance among experimental groups was determined using the one-way or two-way ANOVA with Tukey's post hoc test, with a *P* value of <0.05 considered significant. All analyses were performed using GraphPad Prism 10 (GraphPad Software, La Jolla, CA, USA).

## Results

### Silver Nanoformulations Exhibit pH-Dependent Controlled Drug Release

**Figure 1.**
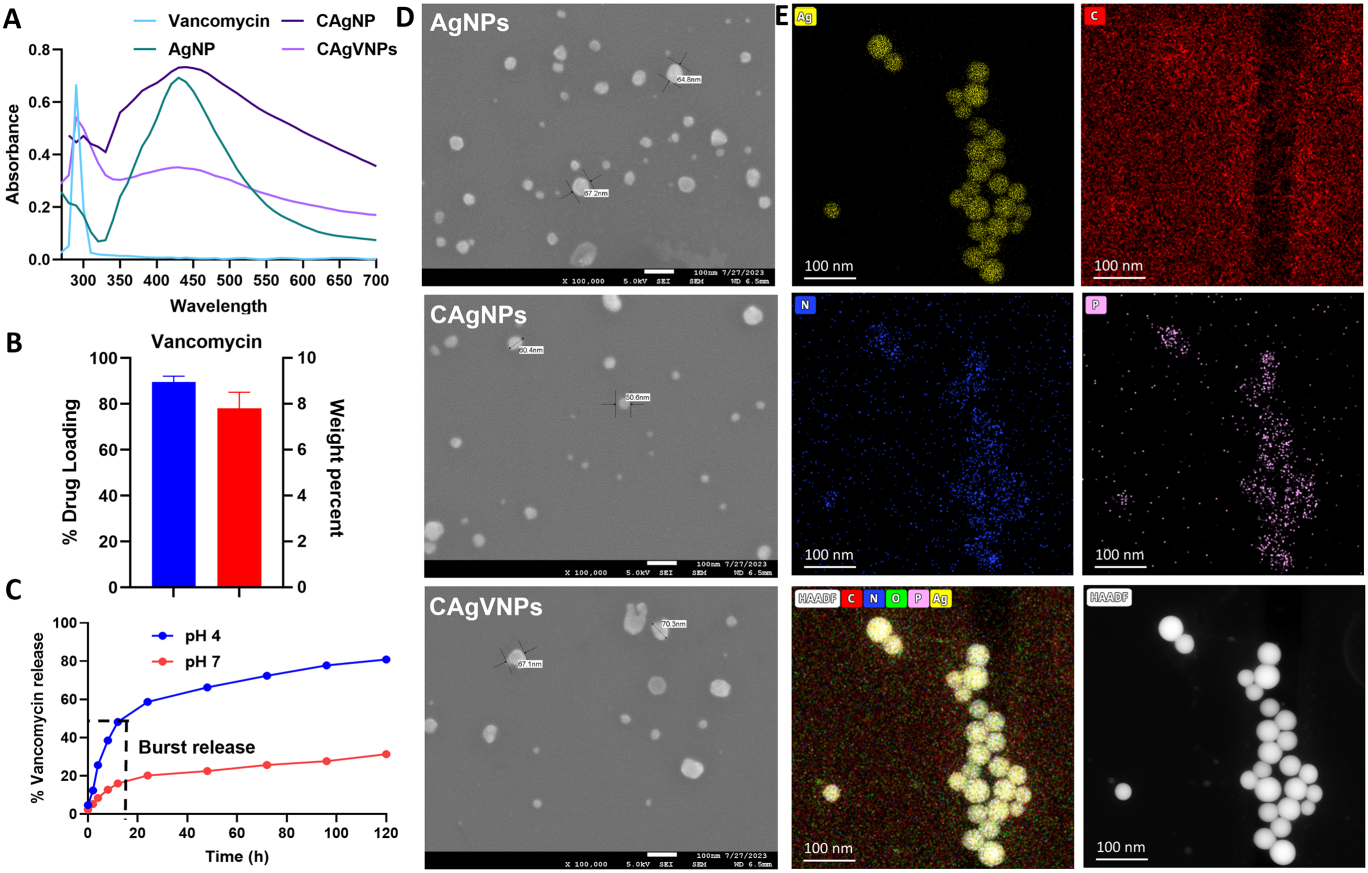
**Characterization of silver nanoparticles (AgNPs).** (**A**) UV-Visible spectra showing the characteristic peaks at 290 nm (vancomycin) and 434 nm (AgNPs). (**B**) Drug-loading efficiency (*left* *axis*) and weight percent (*right* *axis*) of vancomycin on nanoparticles (*n* = 3). (**C**) Drug (vancomycin) release over 120 hours in acidic (pH 4) and neutral (pH 7) conditions (*n* = 3). (**D**) SEM images showing morphology of AgNPs, CAgNPs, and CAgVNPs. (**E**) STEM elemental mapping showing the distribution of silver (Ag), carbon (C), nitrogen (N), and phosphorus (P) elements in CAgVNPs. *Scale*: 1 unit = 100 nm (SEM and STEM).

The silver nanoparticles were synthesized using a chemical reduction method, yielding particles with an average diameter of 58 ± 5 nm and a negative zeta potential (−10.8 ± 3 mV). Next, these nanoformulations were coated with chitosan, resulting in a positive zeta potential (37.8 ± 5 mV) due to the presence of protonated amino groups on the chitosan surface, enhancing their stability. Vancomycin-loaded nanoformulations were synthesized, and Zetasizer depicted a size of 65 ± 7 nm with positive zeta potential (Table 1). The UV spectrum analysis of bare drug, nanoparticles, and drug (vancomycin)–loaded nanoformulations confirmed the presence of respective elements. The peak at 290 nm was observed in bare drug and drug-loaded nanoparticles, whereas the peak at 434 nm was observed in AgNPs, CAgNPs, and CAgVNPs (Fig. 1A). Drug loading in chitosan silver nanoformulations was 89% ± 3% and 9% ± 2% by weight percent (Fig. 1B). The drug release assay demonstrated a distinct pH-dependent pattern. At acidic pH, a burst release of 48% ± 1% was observed within 12 hours, followed by a cumulative release of nearly 81% ± 1% of the drug within 120 hours, indicating a sustained and controlled release. In contrast, at neutral pH (pH 7), the release was lower but stable, with only about 31% ± 1.6% released over the same period (Fig. 1C; Supplementary Table S1).

**Table 1.** Characterization of Nanoformulations

Nanoparticles	Size (nm)	PDI	Zeta Potential (mV)
AgNPs	58 ± 5	0.26 ± 0.05	−10.8 ± 3
CAgNPs	67 ± 5	0.271 ± 0.07	38.2 ± 5
CAgVNPs	65 ± 7	0.214 ± 0.05	37.8 ± 5

Silver nanoparticles and nanoformulations were characterized by the Malvern Zetasizer for size, polydispersity index, and zeta potential. Data are shown as mean ± SD (*n* = 4).

Higuchi constant (K_H_) was calculated to be 10.5 and 3.7 at pH 4 and pH 7, respectively (Supplementary Fig. S2, Supplementary Table S2). Thus, the rate of drug (vancomycin) release from the polymer matrix was higher at acidic pH than at neutral pH. The final formulations were subjected to SEM analysis, which indicated a nanoparticle size of 65 ± 7 nm (Fig. 1D). Furthermore, STEM elemental characterization showed the presence of all metals Ag, C, N, P, and O in the nanoformulation, with silver being the predominant. Traces of phosphorus (P) were contributed by PBS, which was used to rinse and disperse nanoformulations on the copper grids for STEM analysis (Fig. 1E). Together, these results show that our nanoformulation can release antibiotics in a controlled manner.

### Nanoformulations Exhibit Potent Antibacterial Activity Against *S. aureus*

**Figure 2.**
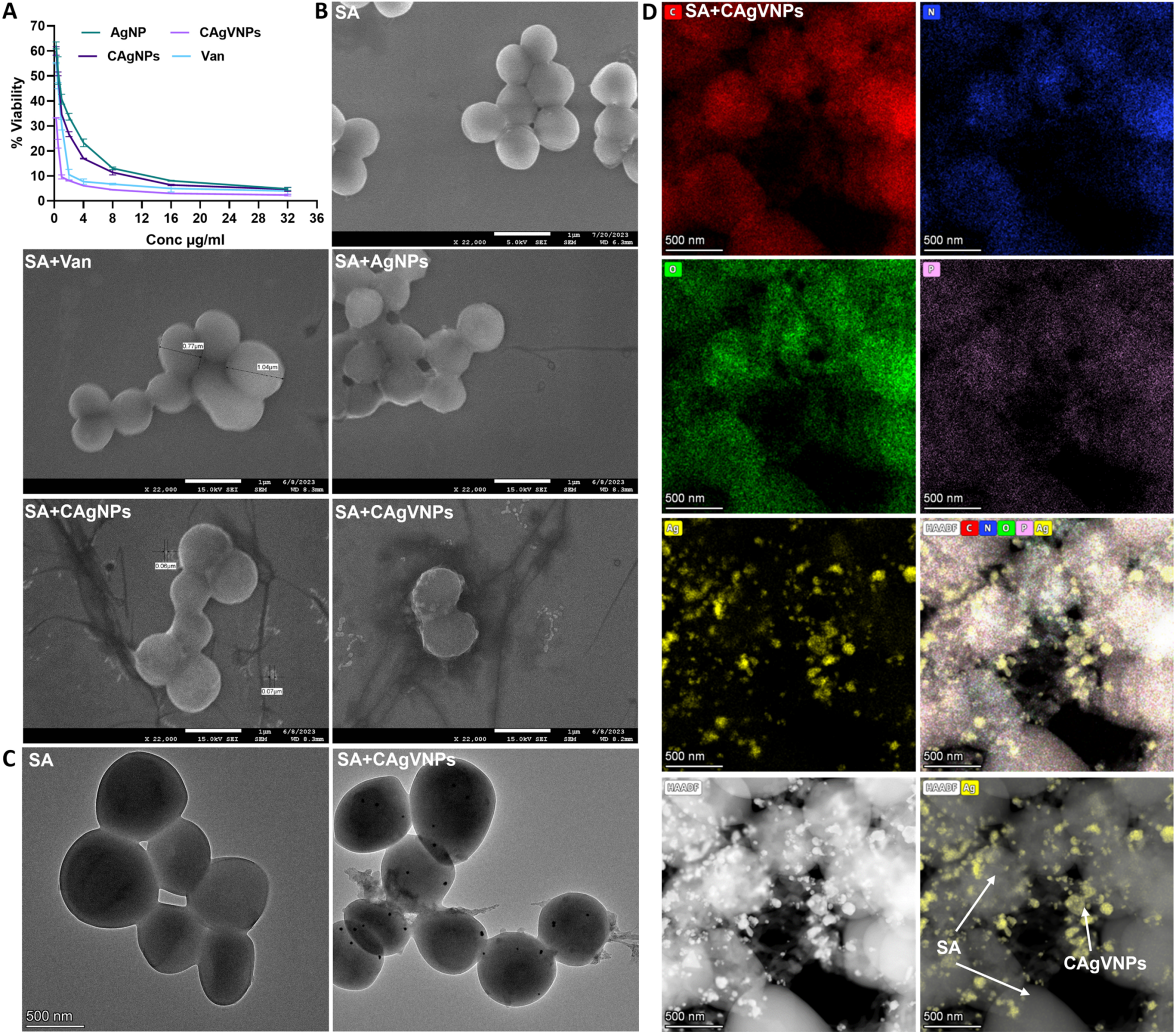
**Assessment of antibacterial activity of nanoformulations.** (**A**) *S. aureus* (strain RN6390) was incubated with increasing concentrations of vancomycin, AgNPs, CAgNPs, and CAgVNPs, and antibacterial activity was assessed by measuring the MIC using bacterial plate count. (**B**) SEM images of *S. aureus* morphology after treatment with different nanoformulations at 0.5 × MIC90. (**C**) TEM analysis comparing untreated and CAgVNPs-treated bacteria. (**D**) STEM elemental mapping showing the distribution of silver (Ag), carbon (C), nitrogen (N), and phosphorus (P) in CAgVNP-treated *S. aureus*. *n* = 3 each experimental condition. *Scale*: 1 µm (SEM), 500 nm (TEM and STEM).

The antimicrobial activity of nanoformulations was tested against the laboratory strain (RN6390) and clinical isolates of SA. Our results show that all nanoformulations significantly reduced MIC-90 as compared to vancomycin alone. Moreover, the antimicrobial activity was enhanced by the positive surface charge, as evidenced by a fourfold reduction in MIC with chitosan-coated nanoparticles (0.25 to 0.5 µg/mL) in comparison to vancomycin (2 µg/mL) alone (Fig. 2A). Similarly, the minimum bactericidal concentration for CAgVNPs was 8 µg/mL, and the bare vancomycin was estimated to be <16 µg/mL.

SEM analysis indicated that chitosan allowed the adherence of the nanoformulations to the bacterial cell wall, whereas silver nanoparticles aided penetration into the bacteria, resulting in leakage of cytoplasm, causing SA lysis (Fig. 2B). The TEM analysis further confirmed that CAgVNPs punctured the SA cell wall, allowing the cytoplasm to spill over (Fig. 2C). The STEM analysis revealed the presence of the silver element on the bacterial surface and within bacteria, showing the distribution and activity of nanoformulations (Fig. 2D). The phosphorus detected was contributed by the phosphates in the bacterial cell wall. Next, the antibacterial activity of nanoformulations was tested against clinical isolates of SA from patients with endophthalmitis, showing their effectiveness at two- to fourfold lower MIC, including USA300, a methicillin-resistant *S. aureus* (MRSA) strain (Table 2; Supplementary Fig. S3).

**Table 2.** Antibacterial Activity (MIC-90) of Nanoformulations Against Clinical Isolates and USA300

SA Isolates	Van (µg/mL)	AgNPs (µg/mL)	CAgNPs (µg/mL)	CAgVNPs (µg/mL)
E442	1	4	1	0.25
E427	2	4	2	0.5
E475	1	4	2	0.5
E331	2	4	4	1
E311	2	4	4	1
USA300	4	4	2	1

Clinical isolates and USA300 strains of *S. aureus* were subjected to varying concentrations of nanoformulations and vancomycin. MIC-90 (µg/mL) values were calculated based on bacterial viability.

### Nanoformulations Exhibit No Toxicity Toward Retinal Cells

**Figure 3.**
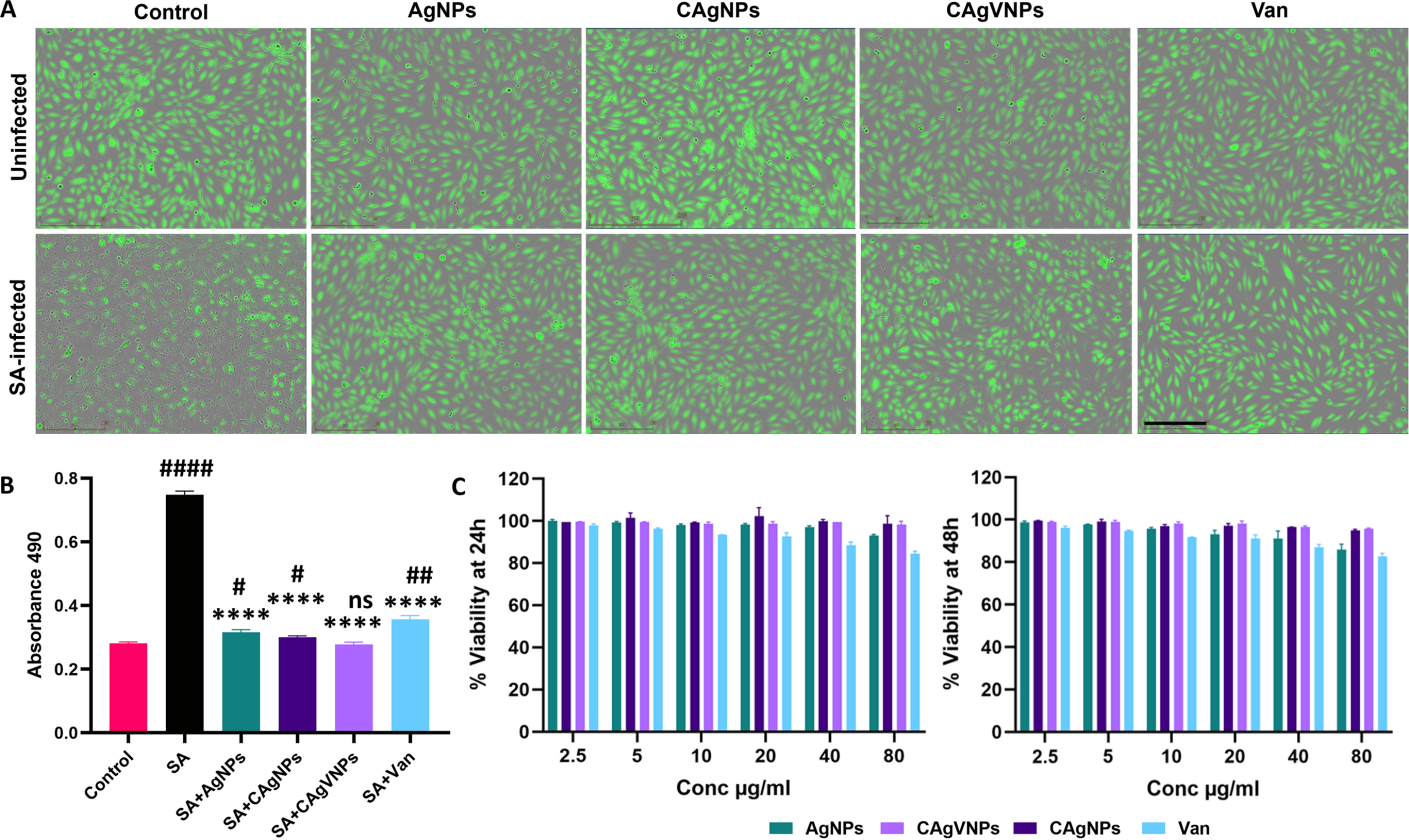
**Evaluation of in vitro toxicity of nanoformulations.** Human retinal Müller glia (MIO-M1 cell line) were left uninfected or infected with *S. aureus* (MOI = 10:1) for 2 hours, followed by treatment with nanoformulations for 6 hours. (**A**) Live-cell imaging was performed using the IncuCyte system, and representative fluorescence (calcein AM stain, *green*) images were captured to show cell survival in response to indicated treatments. (**B**) The effect of various nanoformulations or antibiotic (vancomycin) on *S. aureus*–induced cell death was assessed by measuring LDH release in the culture supernatant. (**C**) Alamar blue assay was performed by incubating MIO-M1 cells for 24 or 48 hours with various nanoformulations to further assess cytotoxicity. Data were tested for normality using the Shapiro–Wilk test and analyzed by one-way ANOVA with Tukey's multiple comparisons. ****P < 0.0001, as compared to SA; ^####^*P* < 0.0001, ^##^*P* < 0.01, ^#^*P* < 0.05; ns, no significant difference as compared to control; *n* = 3 each experimental condition. *Scale*: 500 µm (live-cell imaging).

Müller glia are the major glial cell type in the retina,^43^ and our prior studies have demonstrated their protective role in preserving retinal integrity during endophthalmitis.^44^^,^^45^ Therefore, we used these cells (MIO-M1 line) to evaluate the cytotoxicity of our nanoformulations. Silver nanoformulations were tested for toxicity in the presence or absence of SA infection using calcein-AM dye (green, fluorescent) in a live-cell imaging system (IncuCyte). As anticipated, SA infection significantly reduced calcein-positive (Fig. 3A) cells, indicating increased cell death compared to uninfected control cells. Notably, treatment with all nanoformulations significantly rescued cell viability during SA infection and exhibited no apparent toxicity. To further assess cytotoxicity, we performed an LDH release assay and observed that SA-infected cells exhibited threefold higher LDH levels than uninfected control and nanoformulation-treated cells (Fig. 3B). The AlamarBlue assay further confirmed the lack of significant toxicity when tested at various concentrations for 24 and 48 hours (Fig. 3C). These results demonstrate that nanoformulations do not exert toxicity in vitro.

### Nanoformulations Are Internalized and Exert Intracellular Bacterial Killing

**Figure 4.**
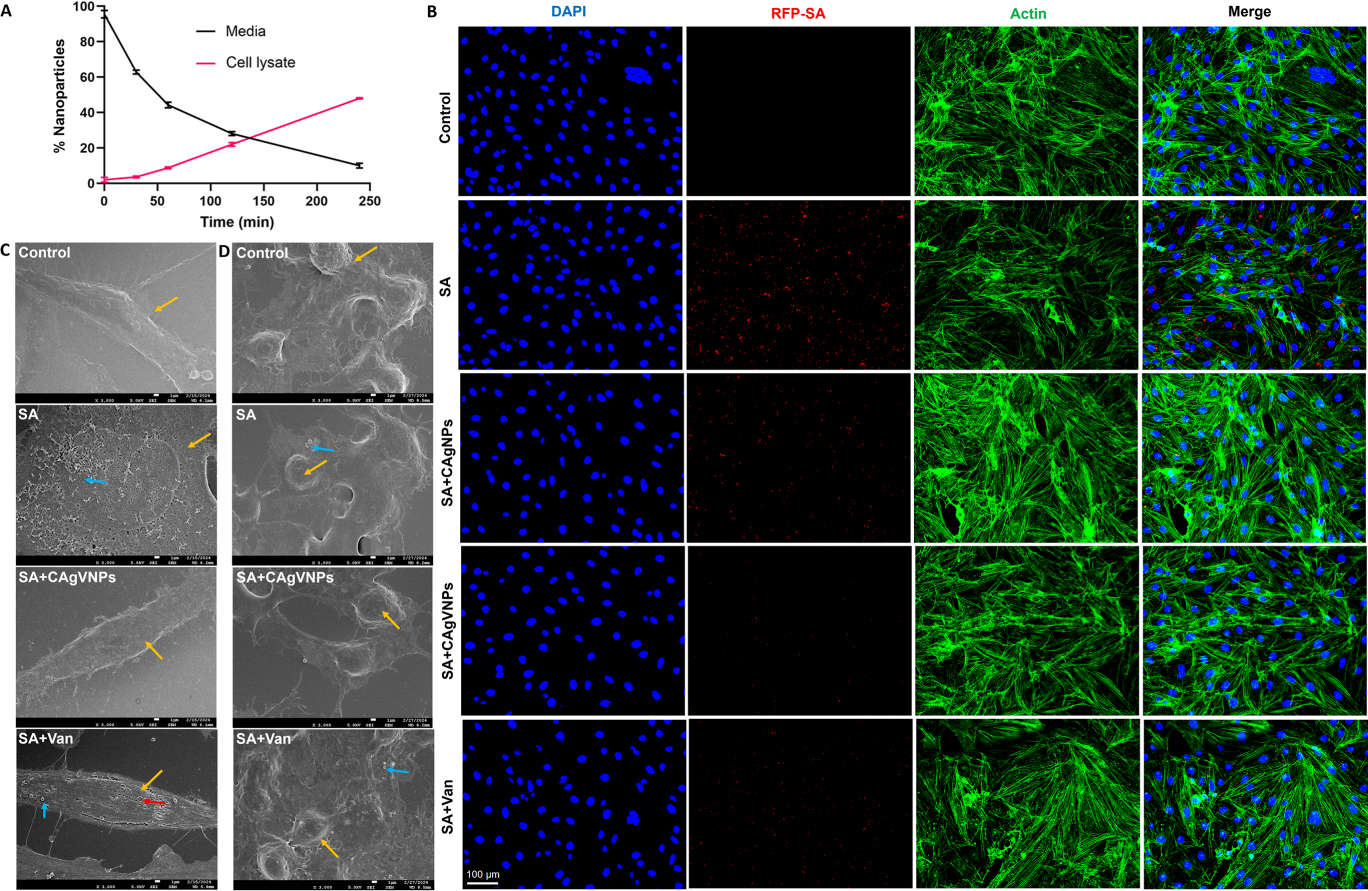
**Cellular uptake and intracellular antibacterial activity of nanoparticles.** (**A**) Human retinal Müller glia (MIO-M1 cell line) were incubated with CAgVNPs, and cellular uptake was measured by assessing nanoparticle concentration in the culture media versus cell lysate at indicated time points up to 240 minutes. (**B**) MIO-M1 cells were infected with RFP-tagged *S. aureus* (RFP-SA, *red*) to visualize intracellular bacteria and assess the antibacterial activity of indicated nanoformulations. Immunohistochemistry analysis shows DAPI-stained nuclei (*blue*), actin (*green*), and RFP-SA (*red*), with merged images showing reduced RFP levels in the treatment groups. SEM analysis of (**C**) Müller glia and (**D**) mouse iBMDMs, showing morphologic changes associated with the internalization of *S. aureus* and the effects of CAgVNP and vancomycin treatments. Arrow color: host cells (*yellow*), *S. aureus* (*blue*), and cell surface pores (*red*). *Scale*: 1 µm (SEM), 100 µm (fluorescent images), *n* = 3.

*S. aureus* can be internalized by immune cells such as macrophages. We next checked whether our nanoformulation could be taken up by cells and exerted intracellular antimicrobial activity. MIO M1 cells were pretreated with nanoformulations, and the concentration (absorbance at 434 nm) of silver nanoparticles in the media and the cell lysate was assessed at different time points. Our data showed that nanoparticle levels decreased in the media but temporally increased in cell lysates, indicating their internalization (Fig. 4A). To further check the internalized nanoparticles and their antibacterial activity, nanoparticles (CAgVNPs) were tested against RFP-SA and visualized under fluorescent microscopy. These nanoparticles reduced RFP levels inside the Müller glia, indicating their intracellular antimicrobial activity (Fig. 4B). The cellular interaction of nanoformulations was assessed by SEM analysis of SA-infected and nanoparticle-treated Müller glia (Fig. 4C) and mouse iBMDMs (Fig. 4D). The control, uninfected Müller cells showed normal cellular structures; however, the SA-infected cells became rounded and underwent lysis with multiple pores and adherence of SA colonies. Similar changes were observed in SA-infected iBMDMs, with SA trying to invade the cell. In contrast, the CAgVNPs and vancomycin treatments rescued cells.

### Nanoformulations Exert Antimicrobial Activity in Ex Vivo Models of Ocular Infections

**Figure 5.**
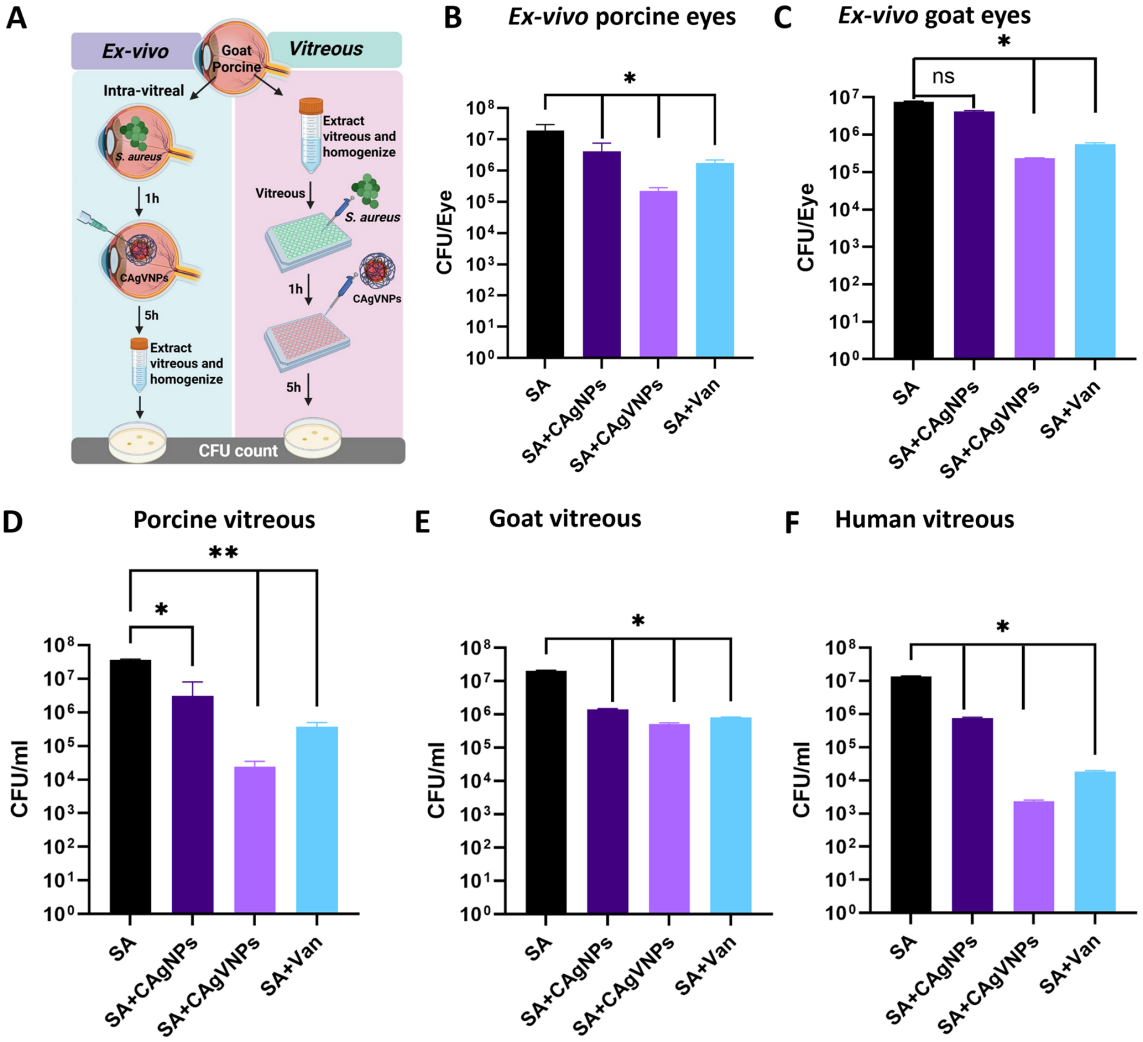
**Antibacterial activity of nanoformulations in ex vivo intraocular infection models.** (**A**) Schematic of ex vivo experiments using goat and porcine eyes and vitreous. (**B**) Goat (*n* = 4) and (**C**) porcine (*n* = 4) eyes were procured from a local slaughterhouse and sterilized by soaking in beta iodine for 5 minutes. Eyes were intravitreally inoculated with 100 µL *S. aureus* containing 5 × 10^6^ CFU. One hour after *S. aureus* infection, eyes were treated with intravitreal injections of CAgVNPs, CAgNPs, or vancomycin (1 µg/mL) for 5 hours, and vitreous was used for CFU enumeration using bacterial plate count. In another set of experiments *S. aureus* was incubated with indicated nanoformulations in the presence of 100 µL vitreous from porcine (**D**), goat (**E**), and human (**F**) eyes (*n* = 3), and bacterial survival was assessed by plate count. Data were tested for normality using the Shapiro–Wilk test and analyzed by one-way ANOVA with Tukey's multiple comparison. **P < 0.01, *P < 0.05; ns, no significant difference.

Next, we tested the efficacy of nanoformulations in ex vivo models to better stimulate the complex ocular environment (Fig. 5A). All nanoformulations demonstrated significant potency in reducing intraocular bacterial burden when tested in ex vivo models using porcine (Fig. 5B) and goat (Fig. 5C) eyes, as well as vitreous humor. Data revealed a marked antibacterial effect, indicating effective intraocular penetration and sustained activity. Importantly, the presence of vitreous, whether porcine (Fig. 5D), goat (Fig. 5E), or human (Fig. 5F), did not diminish the formulation's efficacy, suggesting its potential applicability across species, including in human intraocular environments. Most importantly, the dual-acting combination of vancomycin and silver nanoparticles exhibited synergistic antimicrobial activity, resulting in enhanced bacterial killing compared to either agent alone. Together, these results underscore the therapeutic advantage of these nanoformulations in reducing intraocular bacterial burden.

### Treatment With Nanoformulations Ameliorate SA Endophthalmitis in Mice

**Figure 6.**
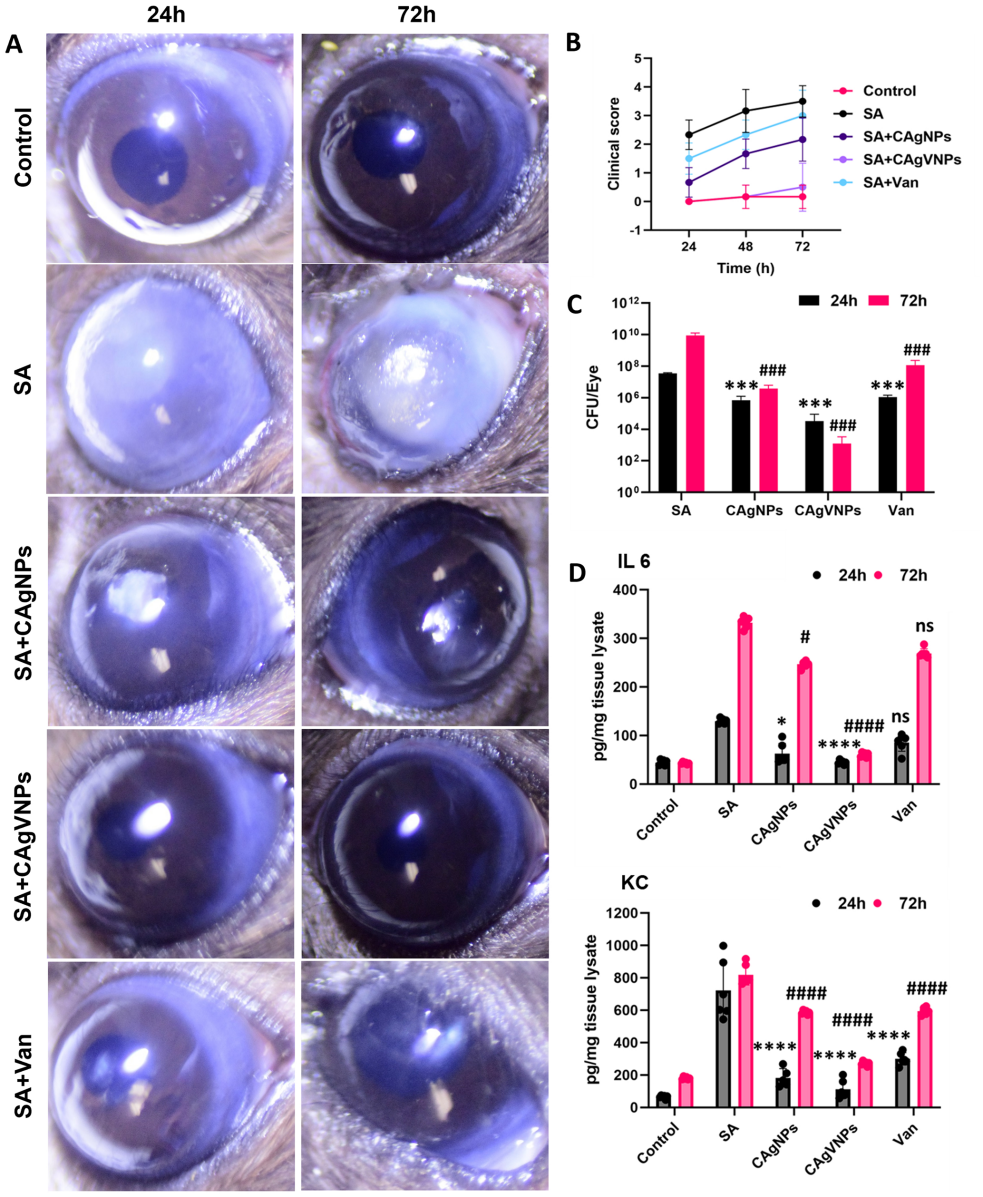
**Therapeutic efficacy of nanoformulations in experimental bacterial endophthalmitis.** Endophthalmitis was induced in C57BL/6 mice (*n* = 6/group) by intravitreal inoculation of *S. aureus* (RN6390; 5000 CFU/eye). Six hours postinfection, mice received intravitreal injections of nanoformulations (CAgNPs or CAgVNPs) or vancomycin at a concentration of 0.2 µg/µL. Disease severity was evaluated by eye exam using slit lamp (**A**) and assigning clinical scores (**B**) based on corneal haze/opacity (ranging from 0 to 4, with 0 being the best and 4 being the worst) at 24 and 72 hours posttreatment. (**C**) Bacterial burden was assessed by serial dilution and plate counting of whole-eye lysates (*n* = 6). The same whole-eye lysates (equal protein concentration) were used to quantify IL6 and KC using ELISA (**D**). Data were tested for normality by the Shapiro–Wilk test, followed by analysis by two-way ANOVA with Tukey's multiple comparisons. ****P < 0.0001, ***P < 0.001, *P < 0.05 (compared to SA infection at 24 hours) and ^####^*P* < 0.0001, ^###^*P* < 0.001, ^#^*P* < 0.05 (compared to SA infection at 72 hours); ns, no significant difference.

**Figure 7.**
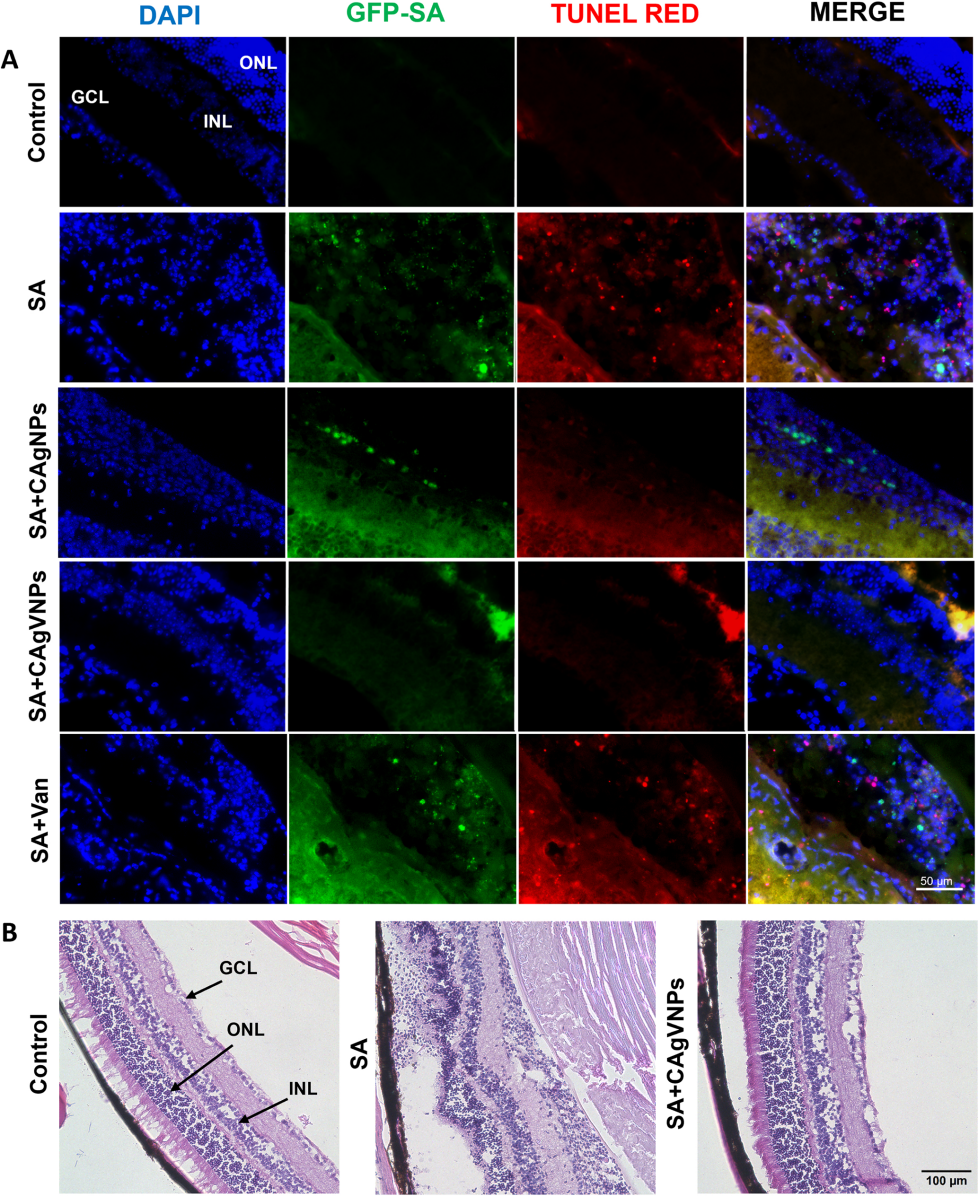
**Effect of nanoformulations on retinal cell death during bacterial endophthalmitis.** Endophthalmitis was induced in C57BL/6 mice (*n* = 5/group) by intravitreal inoculation with GFP-expressing *S. aureus* (SA, 5000 CFU/eye). Six hours postinfection, mice received intravitreal injections of CAgVNPs or vancomycin at a concentration of 0.2 µg/µL. At 24 hours posttreatment, eyes were fixed in paraformaldehyde and embedded in OCT, and 10-µm cryosections were subjected to (**A**) TUNEL staining (*red*), *S. aureus* (GFP), and nuclear counterstaining with DAPI (*blue*). (**B**) Hematoxylin and eosin (H&E) staining was performed in some representative sections to visualize histological changes. GCL, ganglion cell layer; INL, inner nuclear layer; ONL, outer nuclear layer. *Scale bars*: 50 µm (TUNEL); 100 µm (H&E).

Given the potent antibacterial activity of the nanoformulations against *S. aureus* in vitro and ex vivo studies, we next evaluated their efficacy in vivo using a mouse model of *S. aureus* endophthalmitis. Briefly, mouse eyes were treated with intravitreal injections of vancomycin or nanoformulations at 6 hours post-SA infection and analyzed up to 72 hours posttreatment. As anticipated, SA infection increased opacity and hypopyon formation; all nanoformulation-treated eyes ameliorated endophthalmitis (Fig. 6A), as evidenced by a significant reduction in clinical scores. Among the nanoformulations, the dual-acting nanoparticles, CAgVNPs, exerted better protection compared to CAgNP and vancomycin (used at sub-MIC level) alone (Fig. 6B) at all time points. Eyes were processed at the early (24 hours) and late (72 hours) stages of infection for various assays. The analysis of bacterial burden showed that CAgVNP-treated eyes had threefold less bacterial burden compared to untreated eyes at 24 hours, and this difference became sevenfold at 72 hours (Fig. 6C), indicating an extended antimicrobial effect of the treatment. Similarly, the levels of selected inflammatory mediators (IL-6 and KC) were significantly reduced in nanoformulation-treated eyes at 24 and 72 hours (Fig. 6D), with CAgVNPs showing relatively more reduction. Next, we performed TUNEL staining to assess the effect of treatments on retinal cell death. Our data showed intact retinal layers in control, uninfected eyes, whereas significant retinal folding and cell death (as indicated by TUNEL-positive cells) were observed in SA-infected eyes. In contrast, all nanoformulation treatments reduced TUNEL staining (Fig. 7A). Histological analysis of CAgVNP-treated eyes showed preserved retinal architecture compared with SA-infected and untreated eyes (Fig. 7B).

Because CAgVNPs demonstrated the greatest efficacy in ameliorating SA endophthalmitis, we evaluated their effects following intravitreal administration. CAgVNP did not produce detectable adverse ocular effects, as evidenced by the absence of corneal haze/opacity or hypopyon. Consistent with these observations, no increase in TUNEL-positive cells or the induction of inflammatory mediators was detected up to 72 hours compared to untreated control eyes (Supplementary Fig. S4). Collectively, these results indicate that silver nanoformulation does not induce intraocular toxicity at the use concentrations and that treatment with dual-acting nanoparticles (CAgVNPs) reduces the severity of endophthalmitis.

## Discussion

The treatment of ocular infections such as bacterial endophthalmitis remains a significant clinical challenge due to the rapid progression of the disease, limited intraocular drug penetration, and the growing threat of antimicrobial resistance.^46^ In this study, we demonstrated that a dual-acting nanoparticle formulation containing both silver and vancomycin exhibits superior antimicrobial efficacy compared to vancomycin alone. This combinatorial approach leverages the broad-spectrum antimicrobial properties of silver^47^^,^^48^ and the targeted activity of vancomycin^49^ against gram-positive pathogens, offering a synergistic effect that reduces bacterial burden in the eye. Our findings underscore the therapeutic potential of nanotechnology-based drug delivery systems for the effective management of severe intraocular infections. These nanoparticle formulations not only improve drug stability and bioavailability but may also reduce the required antibiotic dose, thereby minimizing systemic toxicity and the risk of resistance.^50^ Overall, our study represents a significant step forward in the development of innovative ocular therapies that could complement and enhance existing treatments for intraocular infections, such as bacterial endophthalmitis.^51^

The eye is an immune-privileged organ with physical and physiological barriers that protect it from systemic insults, including bloodborne pathogens. One of the most important of these is the blood–ocular barrier, which consists of the blood–aqueous^52^ and blood–retinal barriers.^53^ While this specialized system is vital for maintaining ocular homeostasis and shielding the eye from systemic infections, it also presents a major challenge for therapeutic intervention,^54^ as it restricts the penetration of systemically administered antibiotics into intraocular tissues.^55^ Consequently, systemic antibiotic therapy often fails to achieve effective drug concentrations within the eye, particularly in the posterior segment. This pharmacologic limitation necessitates the use of local delivery methods, such as topical administration for anterior segment infections and intravitreal injection for sight-threatening conditions like endophthalmitis.^56^ These approaches bypass systemic circulation, allowing for higher intraocular drug levels, but they come with potential complications such as repeated intravitreal injection-associated endophthalmitis, highlighting the need for improved drug delivery systems and adjunctive therapeutic strategies.

Recently, nanoparticle-based drug delivery systems have been extensively studied for their ability to cross the blood–brain and blood–retinal barriers and for the treatment of various brain and eye diseases.^57^^,^^58^ However, their applications for the treatment of ocular infections have been limited. Nano-sized lipid and polymeric nanoparticles (NPs) 1 to 100 nm in size have been reported to have better permeability across biological membranes, which can increase the drug bioavailability and retention time in ocular tissues.^59^^,^^60^ Here, we made silver nanoparticles due to their inherent antibacterial properties. Silver nanoformulations consistently release silver ions that penetrate both the cell wall and plasma membrane, ultimately lysing the bacteria to exert their antimicrobial effects.^59^^,^^60^ Since the bacterial cell wall is negatively charged, we coated our nanoparticles with chitosan to make them positively charged. The chitosan coating enhanced the adherence of the silver nanoformulations to the *S. aureus* surface and facilitated their penetration. Both TEM and SEM imaging confirmed bacterial membrane disruption, showing direct puncturing and intracellular localization of the nanoparticles. We also evaluated the antimicrobial activity of nanoformulations against *Pseudomonas aeruginosa* and *Candida albicans*, two additional pathogens that can cause ocular infections. We observed the disruption of their cell walls by CAgNP treatment (data not shown), suggesting broad-spectrum potential. Collectively, these findings support the therapeutic potential of silver-based nanoformulations as a targeted, non-antibiotic adjunct therapy for the treatment of ocular bacterial and fungal infections.

The current standard treatment for bacterial endophthalmitis relies on the intravitreal injection of vancomycin and ceftazidime to provide broad-spectrum empirical coverage against both gram-positive and gram-negative pathogens.^6^^,^^61^ However, this approach faces several critical challenges. Vancomycin, while effective against gram-positive bacteria such as *S. aureus*, has been reported to cause toxicity^62^ and potential side effects such as hemorrhagic occlusive retinal vasculitis at higher or repeated doses.^63^ In addition, the rising incidence of antibiotic resistance, including reduced susceptibility of MRSA to vancomycin, further undermines its effectiveness.^64^ Based on these limitations, we hypothesized that using a lower dose of vancomycin when combined with our silver nanoformulations could retain or even enhance antimicrobial efficacy while minimizing potential ocular toxicity. Our nanoformulations not only demonstrated potent antibacterial activity against clinical endophthalmitis and MRSA isolates but also showed no toxicity in cultured retinal Müller glia and mouse eyes. Notably, our chitosan–silver–vancomycin nanoparticles (CAgVNPs) significantly reduced vancomycin's MIC, demonstrating a synergistic effect. This combinatorial approach presents a promising strategy for safer and more effective treatment of endophthalmitis. One of the unique advantages of our nanoformulations is their capacity to not only adhere to and disrupt bacterial membranes but also to enter retinal cells. This intracellular access allows the nanoformulations to target and eliminate bacteria residing within retinal cells, a reservoir that is typically inaccessible to most conventional antibiotics.^65^ This property significantly reduces the risk of chronic or relapsing infection, a complication that is notoriously difficult to manage with standard therapies alone.^66^

Another important aspect of our nanoformulation is its pH-dependent drug release behavior. Bacterial infection is known to lower local tissue pH, creating an acidic microenvironment.^67^^,^^68^ Because our nanoformulation incorporates chitosan, the polymeric matrix remains compact and stable under physiological (neutral) pH conditions but undergoes structural relaxation at acidic pH, resulting in enhanced drug release.^18^^,^^69^ Consistent with this mechanism, our release studies show an accelerated early release of vancomycin under acidic conditions, followed by sustained release over time, whereas drug release was significantly lower at neutral pH. This pH-responsive behavior closely mimics the intraocular infectious milieu and supports targeted antibiotic delivery at sites of infection while limiting release in healthy tissue.

Next, we investigated whether the antimicrobial efficacy of our nanoformulations is maintained within the intraocular environment. To mimic clinical conditions, we conducted ex vivo experiments using porcine and caprine eyes and human vitreous samples. These models allowed us to assess the performance of our nanoformulations in a physiologically relevant setting that closely resembles the ocular microenvironment during infection.^70^ Our data demonstrated a significant reduction in *S. aureus* bacterial burden across all vitreous types, indicating that the nanoformulations retain potent antibacterial activity within the eye. These findings also align and support the prior studies showing the antibacterial activity of silver nanoparticles during *S. aureus* infection.^71^

Finally, our in vivo findings in the *S. aureus* endophthalmitis mouse model highlight the potential of CAgVNPs as a next-generation therapeutic strategy. By enabling effective bacterial clearance while minimizing inflammatory damage and retinal cell death, these nanoformulations address several key limitations of current treatment. Conventional intravitreal antibiotics, especially vancomycin, are limited by their inability to penetrate host cells or neutralize intracellular pathogens that contribute to treatment failure and recurrent infection.^66^ Prior studies have demonstrated that intracellular persistence of *S. aureus* can lead to chronic inflammation despite antibiotic therapy.^65^ Our data suggest that nanoformulation-mediated delivery not only enhances antibacterial potency at lower vancomycin doses but also supports tissue preservation, likely due to nontoxicity. This is consistent with emerging evidence that silver and chitosan nanoparticle-based antimicrobial systems can be used for the treatment of eye diseases, including infections.^72^^–^^74^ Moreover, the reduced inflammation and retinal tissue preservation observed in our study align with our previous reports showing that minimizing host tissue damage is critical for long-term visual outcomes in endophthalmitis.^75^^–^^77^ Consistent with our findings, a recent study demonstrated the immunomodulatory role of silver nanoparticles through suppression of activated microglia and inflammatory mediators in the retina.^78^ Together, these findings support the concept that silver nanoparticles can attenuate pathological retinal inflammation, including during infectious endophthalmitis, without compromising retinal functions.

## Conclusions

**Figure 8.**
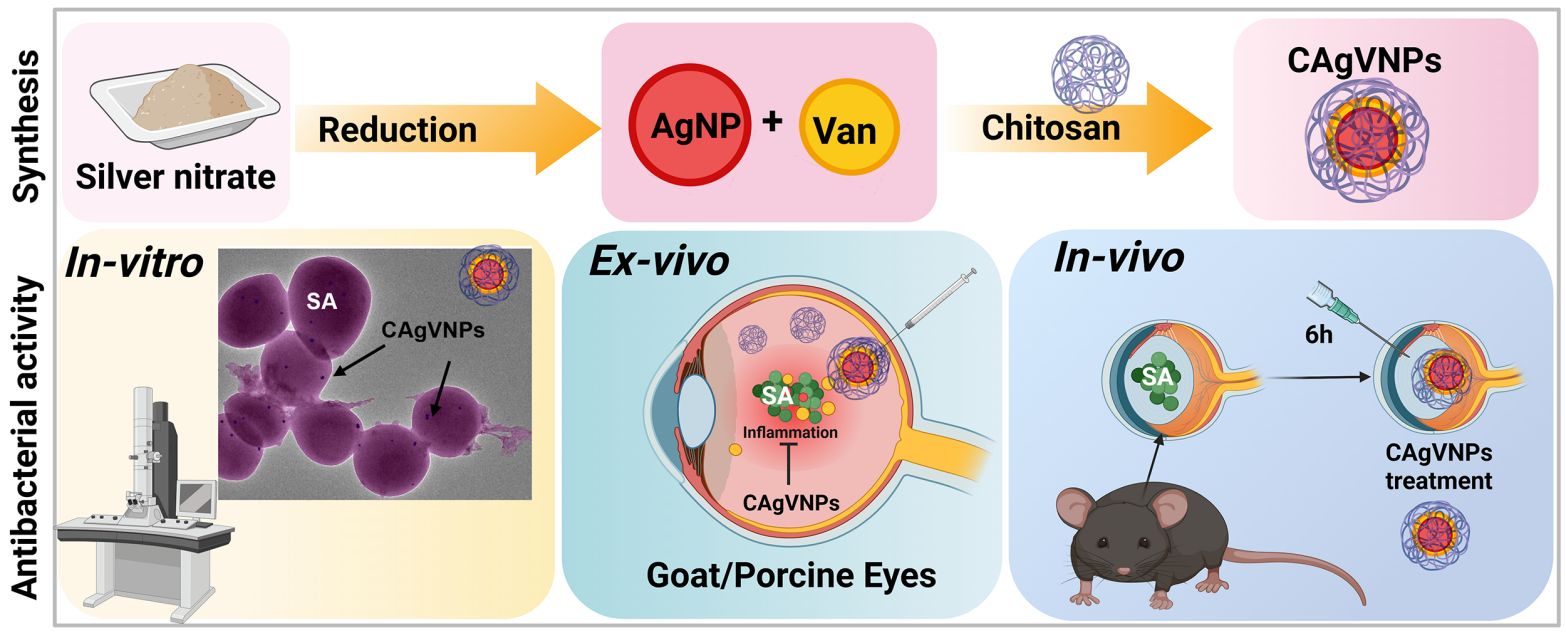
**Schematic of synthesis and evaluation of antibacterial activity of silver nanoformulations.** Silver nanoparticles were synthesized by the chemical reduction method from silver nitrate. Vancomycin was loaded on silver nanoparticles and entrapped in chitosan to generate chitosan-coated, vancomycin-loaded silver nanoparticles (CAgVNPs). The nanoformulations were tested for their antibacterial activity in vitro, ex vivo, and in vivo (mouse model).

We developed and evaluated chitosan-coated silver–vancomycin nanoparticles as a therapeutic modality for the treatment of bacterial endophthalmitis. These nanoformulations demonstrated potent antibacterial activity both ex vivo and in vivo, significantly reducing intraocular bacterial burden while preserving retinal architecture (Fig. 8). By leveraging the synergistic action of silver nanoparticles and reduced doses of vancomycin, our nanoformulations achieved improved efficacy with lower toxicity. Together, our results offer a promising alternative for treating ocular infections, particularly in an era of rising antibiotic resistance and increased emphasis on tissue-targeted, biocompatible therapeutics. However, further studies are needed to fully characterize the in vivo pharmacokinetics of these nanoformulations, including their retention and distribution within the eye, and a head-to-head comparison with the clinical dose of vancomycin. Such investigations will be important to optimize dosing, assess long-term safety, and better understand the therapeutic potential of these nanoformulations in ocular infections.

## Supplementary Material

Supplement 1
