# Supplementary material for: Chitosan-Coated Silver–Vancomycin Nanoparticles for Treatment of Bacterial Endophthalmitis
Source: Invest Ophthalmol Vis Sci. 2026 Mar 10;67(3):20. doi: 10.1167/iovs.67.3.20 (PMC12988689; doi:10.1167/iovs.67.3.20)
Supplement: Supplement 1 [file iovs-67-3-20_s001.pdf]

Chitosan-coated Silver-Vancomycin Nanoparticles for treatment of bacterial endophthalmitis

Authors: Henry Kolge[1], Zeeshan Ahmad[1], Sukhvinder Singh[1], Michael Yu[2], and Ashok Kumar[1, 3]*

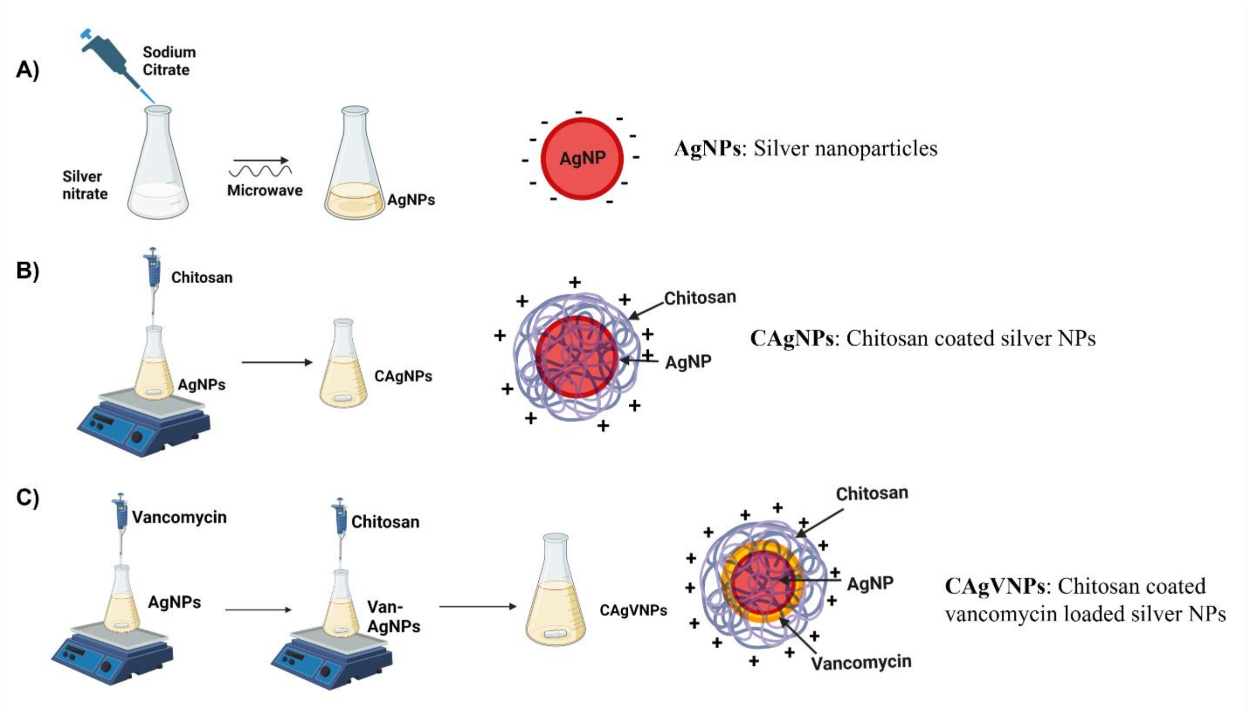

Fig. S1: Schematic of nanoformulation synthesis strategy: A) Silver nanoparticles (AgNPs) were synthesized via chemical reduction, in which silver nitrate (0.5 µM) was reduced in the presence of sodium citrate (0.5 µM) in the microwave. **B)** Chitosan-coated silver nanoparticles (CAgNPs) were synthesized by adding a chitosan solution (0.5 mg/mL in 0.1 M acetic acid) dropwise to AgNPs under stirring for 1 h. **C)** Chitosan-coated, vancomycin-loaded silver nanoparticles (CAgVNPs) were prepared by mixing vancomycin (5 mg/mL) with the silver nanoparticle solution, followed by dropwise addition of chitosan (0.5 mg/mL) to facilitate drug entrapment.

Table S1: Cumulative release (%) of drug at pH 4 and 7 at different time points.

Time (h)	Cumulative release (pH 4)	Cumulative release (pH 7)
0	4.74±0.45	2.37±0.53
2	12.34±0.52	5.327±0.37
0	25.6±1.2	8.465±0.92
8	38.54±1.45	12.825±1.1
**12**	**48.22±1.1**	16.07±133
24	58.74±0.1.31	20.17±1.03
48	66.27±0.78	22.525±0.97
72	72.385±1.12	25.675±1.51
96	77.79±1.04	27.72±0.67
120	80.85±1.1	31.4±1.28

Drug release showing a burst release (**bold**) followed by controlled release pattern.

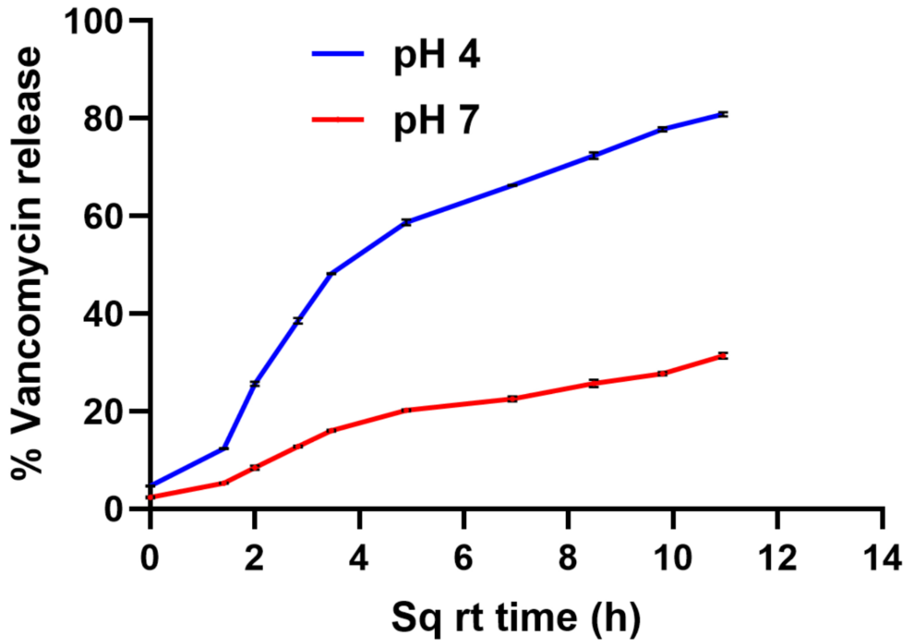

Fig. S2: The Higuchi was applied by plotting cumulative drug release as a function of the square root of time and expressed as $Q=K_H \times t1/2$, where K_H is the Higuchi dissolution constant, and Q represents the cumulative percentage of drug release at time. Data are presented as mean values (*n* = 3).

Table S2: Parameters of the Higuchi model of drug release (R^2 and K_H)

Parameters	pH 4	pH 7
R^2	0.9137	0.9572
K_H	10.498±2.5	3.696±0.69

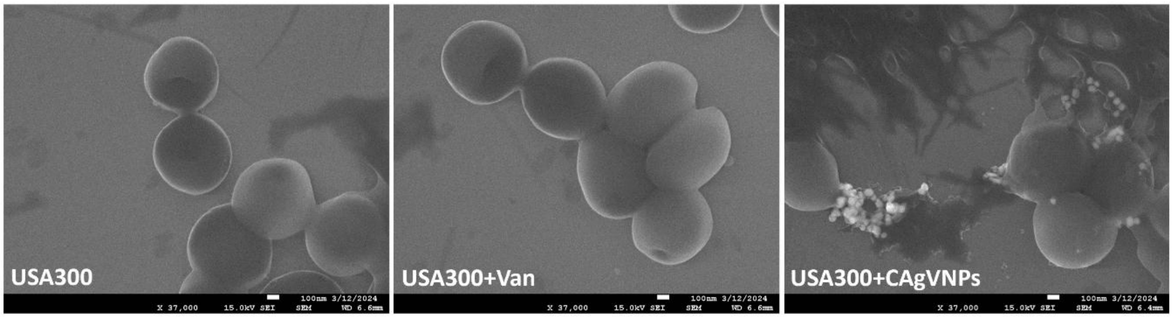

Fig S3: SEM analysis of USA 300 *S. aureus* treated with nanoformulations: USA300 (methicillin-resistant) strain of S. aureus was exposed to vancomycin (van) or CAgVNPs at MIC-90 concentration (1µg/ml) for 24h. Samples were PFA-fixed, followed by ethanol-based serial dehydration, and examined under SEM to visualize antibacterial activity. Scale: 100nm (n=3).

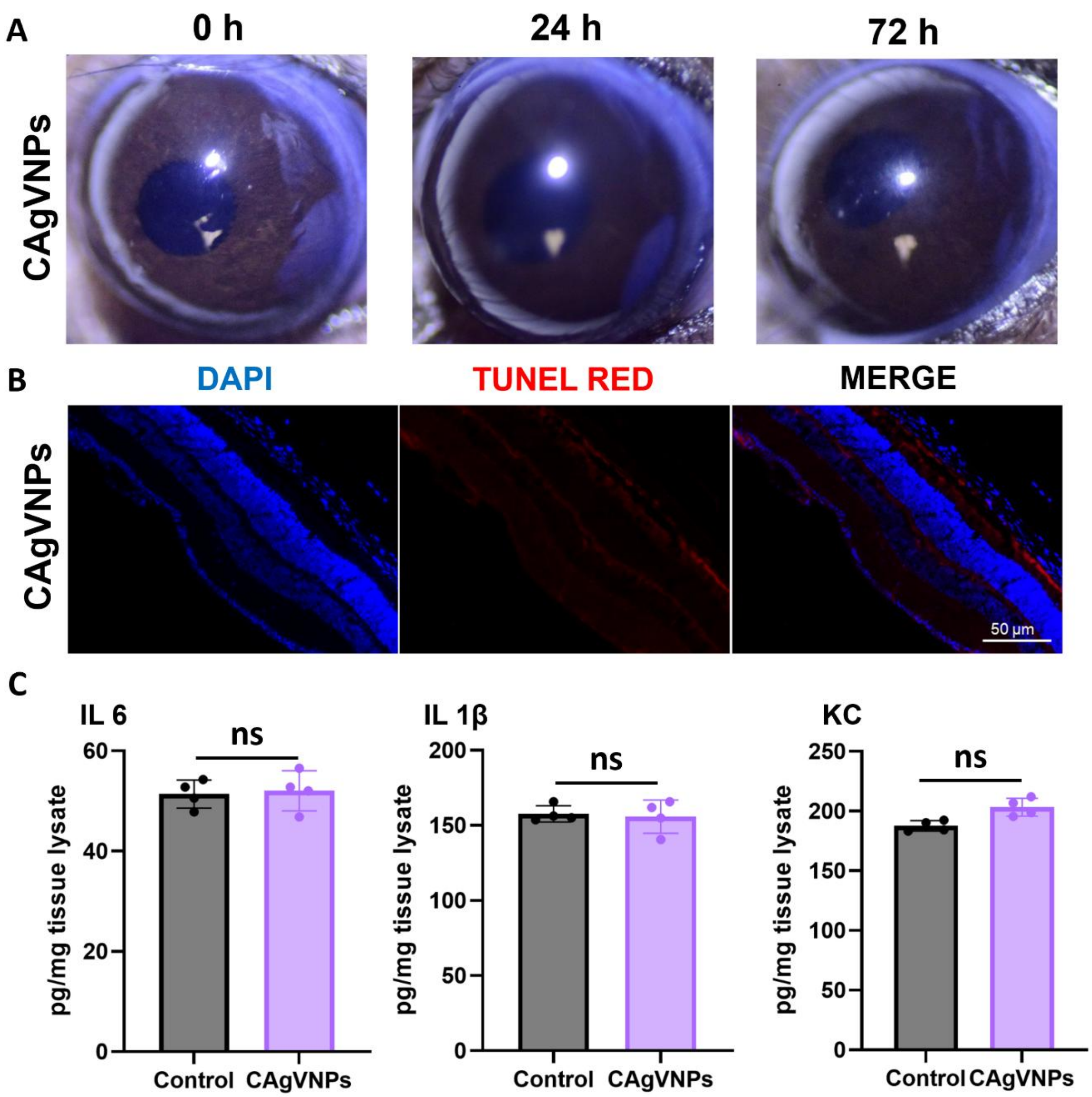

Fig. S4: In vivo toxicity of nanoparticles in mouse eyes: Nanoformulations (CAgVNPs, 0.2 μg/μL per vancomycin concentration) were intravitreally injected in C57BL/6 mouse eyes (n=4). **A).** Representative slit-lamp micrograph showing corneal haze/opacity at 24 and 72 h post-drug treatment. **B)** TUNEL staining (red) of retinal tissue with DAPI (blue). **C)** Whole eye lysate was used to quantify inflammatory cytokines using ELISA, and data are normalized to total protein levels. Data were tested for normality using the Shapiro-Wilk test and analyzed by student t-test. ns, no significant difference. Scale: 50μm.
